# Young women and limits to the normalisation of condom use: a qualitative study

**DOI:** 10.1080/09540120802301857

**Published:** 2009-05-14

**Authors:** Lisa M. Williamson, Katie Buston, Helen Sweeting

**Affiliations:** MRC Social and Public Health Sciences Unit, Glasgow, UK

**Keywords:** condoms, norms, young women, STI prevention, sexual behaviour

## Abstract

Encouraging condom use among young women is a major focus of HIV/STI prevention efforts but the degree to which they see themselves as being at risk limits their use of the method. In this paper, we examine the extent to which condom use has become normalised among young women. In-depth interviews were conducted with 20 year old women from eastern Scotland (*N* = 20). Purposive sampling was used to select a heterogeneous group with different levels of sexual experience and from different social backgrounds. All of the interviewees had used (male) condoms but only three reported consistent use. The rest had changed to other methods, most often the pill, though they typically went back to using condoms occasionally. Condoms were talked about as the most readily available contraceptive method, and were most often the first contraceptive method used. The young women had ingrained expectations of use, but for most, these norms centred only on their new or casual partners, with whom not using condoms was thought to be irresponsible. Many reported negative experiences with condoms, and condom dislike and failure were common, lessening trust in the method. Although the sexually transmitted infection (STI) prevention provided by condoms was important, this was seen as additional, and secondary, to pregnancy prevention. As the perceived risks of STIs lessened in relationships with boyfriends, so did condom use. The promotion of condoms for STI prevention alone fails to consider the wider influences of partners and young women's negative experiences of the method. Focusing on the development of condom negotiation skills alone will not address these issues. Interventions to counter dislike, method failure, and the limits of the normalisation of condom use should be included in STI prevention efforts.

## Introduction

Recently, sexually transmitted infections (STIs), such as *Chlamydia trachomatis*, have increased among young women (The UK Collaborative Group for HIV and STI Surveillance, 2006). Condoms offer protection from these and their promotion is essential to HIV and STI prevention in the UK Sexual Health Strategies (Department of Health, 2001; Scottish Executive, 2005). In the 2000 UK National Survey of Sexual Attitudes and Lifestyles (Natsal, 2000) 80% of 16–19 year olds and 76% of 20–24 year olds reported condom use at first sexual intercourse (Wellings et al., 2001). However, use appears to decrease with age, and in the 2006/07 Office for National Statistics Omnibus Survey only 39% of 20–24 year olds reported current condom use (Lader, 2007).

This paper describes condom use among a sample of young women from eastern Scotland and explores the factors they report are associated with use and non-use of this method. We discuss how, although condom use has, to some extent, been normalised, this is limited by the young women's risk perceptions and actual experiences of use.

## Methods

The findings in this paper come from a qualitative study of young women's patterns of contraceptive use (Williamson, 2007). Interviewees were selected from the SHARE (a randomised trial of a school-based sex education intervention) sample. The intervention did not improve condom use among those who received SHARE sex education (Wight et al., 2002), and we found no differences in the qualitative sample between those in the intervention and control arms of the trial. Ethical approval was granted by the Glasgow University Ethics Committee for Non-Clinical Research Involving Human Subjects.

Purposive sampling was used to select a heterogeneous group of young women at age 20 with different levels of sexual experience and from different social backgrounds: based on father's social class (derived from occupation) and their own educational attainment (both significantly associated with contraceptive use in quantitative analyses of the SHARE data), and area of residence (because of differences in contraceptive services in the main city and rest of study area) (Williamson, 2007). Demographic and sexual experience characteristics of the 20 interviewees are shown in Table 1. The interviewees were evenly split across the sampling frame groups. All but one of the young women was white, reflecting the relatively homogenous ethnic composition of the SHARE sample. The interviewees reported a range of different sexual experiences, but their contraceptive experience was mainly limited to condoms and the pill.

**Table 1:** Basic demographic and sexual experience characteristics of the interviewees.

	Interviewees (*N* = 20)
**Sampling frame characteristics**	
*Sexual experience*	
Two or more sexual partners by age 16	6
First sexual experience by age 16 (one partner)	7
First sexual experience by age 18	7
*Father's social class*	
Manual	10
Non-manual	10
*Educational attainment (at age 16)*	
Credit CSE grades	10
General/foundation CSE grades	10
*Area of residence*	
Main city	9
Rest of study area	11
**Other demographic characteristics**	
*Living arrangements*	
With parents	12
With partner	2
On own with (child/children)	3
Student accommodation	3
*Current employment*	
Working full-time	7
Working part-time	4
Full-time mother	2
In full-time education (college or university)	7
**Sexual experience**	
Age at first sexual intercourse (range)	12–17
Total number of sexual partners (range)	1–16
Boyfriend relationships	20
Casual sex partners	16
Coercive sexual experiences/abusive relationships	4
Pregnancy	8
Tested for STIs	9
Sexually transmitted infection	1
**Experience of contraceptive use (ever use)**	
Condoms	20
Pill	19
Alternatives (e.g. injection)	4
Emergency contraception	16
Non-use (i.e. unprotected sex)	10

* As reported in survey data at age 16 or 18.

† CSE Grades are the exams at the end of statutory education, with credit being the highest level.

‡ As reported at interview at age 20.

The sample is over-representative of those potentially exposed to greatest risk, given that 11 of the interviewees (55%) had first had sex by age 16, compared to 28% of 20–24 year old females in Natsal 2000 (Wellings et al., 2001). In the SHARE survey data collected at age 18, the interviewees reported comparable condom use at most recent sexual intercourse to the rest of the sample (45 and 46%, respectively), but considerably more pregnancy experience (30 and 16%). Also, three of the interviewees (15%) reported motherhood before age 18 compared with only 6% of 20–24 year olds in Natsal 2000 (Wellings et al., 2001).

Most of the interviews took place in the young women's own homes and were conducted in private to ensure confidentiality. They were audio recorded, averaged one hour in length, and covered their sexual, relationship and contraceptive experiences. The interviews were transcribed verbatim and the young women were given pseudonyms.

## Results

### Condom use

All of the young women had used condoms but only three reported always doing so. The rest had changed to other methods, most often the pill, though they typically went back to using condoms occasionally (10 always used condoms with new or casual partners). Most (17) had used a condom at first sexual intercourse, but only five at most recent (two with a new or casual partner and three with a boyfriend).

Factors encouraging condom use included social norms and expectations of condom use and STI prevention, and the accessibility of the method. Those discouraging use were negative experiences of use and expectations of stopping use with boyfriends. Each is considered in turn.

### Reasons for using condoms

#### Social norms and expectations of condom use and STI prevention

Condoms were the first contraceptive method these young women reported using. Most (15) reported some expectation of condom use, particularly when they first had sex or with new or casual partners. The three with strongest expectations thought that it was just what you should do. As a result, they always used condoms:

“I think it's just kind of that's what you just think you're gonnae do, there's not kind of any objections or anything. Cos it's just what you should do …” (Kate).

For this small group, condom use had become normalised and they found it difficult to further justify or explain their use.

Although the social norms of condom use were intrinsically tied to the need for STI prevention, and the condom is the only contraceptive that is also a prophylactic; its contraceptive properties are still seen as most important. The young women talked of being more worried about pregnancy and the STIs they worried about most were those that were easily treatable:

“… HIV and AIDS is not something that I've ever, I've never really considered that I could catch it. … when somebody says like, ‘but what if you catch something?’, the first thing that pops into my mind is like er, chlamydia or genital warts …” (Kim).

For most of the young women, social norms of use centred only on new or casual partners. There was a sense that not using condoms with casual partners was “not the most sensible thing to do”. Casual partners were perceived to pose a greater STI risk because they and their sexual histories were unknown. The need for STI prevention was perceived to be limited to such partners, and the influence of this factor was reduced in relationships with boyfriends. Yet the distinction between casual partners and boyfriends was often tenuous and some said they would use condoms with casual partners but not with partners classed as boyfriends, even when they had relatively short relationships with them.

#### Accessibility

Condom use was reinforced by their accessibility. Although sex education was identified as a source of information on where to get condoms, it had little bearing on use beyond this. Condoms were described as the most obtainable contraceptive method and availability was often cited as the reason for use at first sex:

“… it's like the most readily available, out of any form of contraception, it's condoms really. Em, because you can just buy them anywhere really …” (Milly).

It was also apparent from their accounts that condoms were the only method that partners could provide. Some young women relied on this, and most (11) who used a condom at first sex said that their partner had provided it. However, only four went on to rely solely on their partners for condoms:

“I never carry them, he always carried them. That was his department to look after.” (Margaret).

For many, the decision to use condoms was reported to be a joint one, but relying on partners to supply condoms meant the young women could be subject to pressure not to use them:

“He was trying to be smart and said he only had one condom and deliberately never put it on properly and … he'd says that it had come off and then I stopped him. I knew, I wasnae that daft, I stopped there and then.” (Fiona).

As they got older, most (16) of the young women reported that they carried their own condoms:

“I think because I always had one in my bag and normally … not that you're drunk but I would normally just kinda put it on the bed or throw it at him or, like, well, ‘there you go’ kinda thing. And you know, you see a few guys that are like ‘I'm not using this’. It's like ‘well, you either do or nothing's gonnae happen’ …” (Megan).

Others said they waited to see if their partner would introduce the condom first, and then insisted on use if this did not happen. None suggested that carrying condoms was associated with having a negative sexual reputation.

The accessibility of condoms combined with ingrained expectations of use, meant that they were most often the first contraceptive method the young women used, yet most reported stopping use at some point.

### Reasons for discontinuing condom use

#### Negative experiences of condom use

Fourteen of the young women reported their own, personal dislike of the method, describing how condoms interrupted sex and were “a moment killer”. Condoms also lessened their enjoyment of sex. Sex did not feel as good with condoms because they reduced the sensations:

“I don't know, they just don't feel right. Just something weird about … I don't know, I think it's cos I know that they're there sorta thing. It just doesn't feel right.” (Debbie).

For some, condom use even resulted in sex being painful during or after the event. A minority also reported finding condoms difficult and awkward to use:

“And then all the magazines that tell you they can become a fun part of foreplay. Lies! Lies! They're the worst things. I've been having sex since I was 14 years old and I still can't put one on somebody properly.” (Kathy).

This young woman also went on to talk about how condoms were awkward to dispose of, particularly given that she stayed in shared student accommodation.

It was not inevitable that condom dislike would lead to non-use, and only three reported disliking them so much that they would choose not to have sex rather than to use them. However, the experience of condom failure was more likely to lead to discontinuation. Eight reported condoms breaking, bursting, splitting, or slipping off:

“… we just kept having like condom disasters. They kept coming off and I was just, I think it happened, it must have happened twice …” (Tammy).

When this happened, condoms came to be seen as an ineffective pregnancy prevention method. However, none of the young women reported pregnancy or STIs as a result of condom failure; all reported using emergency contraception but only three had been screened for STIs (and not as a result of the condom failure).

#### Expectations of stopping condom use with boyfriends

Just over half (12) of the young women said they had stopped using condoms within a boyfriend relationship, often when they felt the relationship was well enough established. There was no set timing for this, with condoms being stopped after anything from a few weeks to almost a year. Condom discontinuation was seen as a demonstration of trust:

“… at first we did use contraception [condom] but then a couple of months into the relationship we were like ‘nah, it's alright now’, you know? […] … so basically I had a wee discussion about that and said we don't need to use them any more, we're quite safe wi each other.” (Melanie).

However, only one young woman stopped using condoms *simply* because she was in a relationship. For the rest, this and negative experiences of use were often combined. For example, Melanie, in addition to trusting her boyfriend, particularly disliked the interruption of his stopping to put on a condom. This pattern of condom discontinuation was frequently repeated; if their relationship ended, the young women would start using condoms with their next partner, before again discontinuing use when trust was established. Although they trusted that their boyfriends did not pose an STI risk, they could not always know for sure this was actually the case. Only one young woman went for STI screening before she stopped using condoms with her boyfriend.

## Discussion

Condom promotion is integral to STI prevention in the UK and although many young women report use at first intercourse, it generally decreases over time (Darroch, Singh, Frost, & the Study Team, 2001; Lader, 2007; Wellings et al., 2001). This pattern was evident in the reports of the young women in this study. Condom use was universal, but rarely consistent, and whereas reasons for use were most salient at first sexual intercourse and with new or casual partners, reasons for non-use emerged with experience.

The findings are from a small qualitative sample within one particular geographical locality (eastern Scotland) and caution should be taken generalising beyond this population. Although this should be considered when interpreting our results, our novel finding that condom use has (to some extent) been normalised at first sex and with casual partners, but continued use is limited by negative experiences and dislike of the method has important implications for STI prevention. The following discussion concentrates on this finding (see Williamson, 2007 for further contextualisation and discussion of all of the young women's experiences of condoms and other contraceptive methods).

For a few young women, consistent condom use had become the perceived social norm. Belief in positive social norms of condom use can encourage use (Hatherall, Stone, Ingham, & McEachran, 2005; HEA, 1999; Schaalma, Kok, & Peters, 1993), and there are reports elsewhere of use becoming the norm for some (Coleman, 2001; Lear, 1995; Maharaj & Cleland, 2006). Although, for many it was the partner who supplied condoms at first sex (Mitchell & Wellings, 1998), most went on to carry their own supply (without fear for their reputations) as they got older and more sexually experienced. None suggested this was associated with a negative reputation, contradicting much of the previous literature (Browne & Minichiello, 1994; HEA, 1999; Hillier, Harrison, & Warr, 1998; Holland, Ramazanoglu, Sharpe, & Thomson, 1998; Kirkman, Rosenthal, & Smith, 1998; Lees, 1993; Stewart, 1999). Yet, for most of the young women in this study the normalisation of condom use was limited to new or casual partners. Almost all reported using condoms with their casual partners without question and it can only be surmised that this is a result of the policy initiatives and health promotion campaigns that have followed in the wake of concern over HIV/AIDS and, more recently, STIs in general (Department of Health, 2001; Scottish Executive, 2005). However, most discontinued use once in boyfriend relationships.

As pregnancy prevention was still required, the young women changed to the contraceptive pill at this stage (see Williamson, Buston, & Sweeting, in press), and inability to maintain STI prevention in relationships with boyfriends prevailed. Invulnerability to STIs, the greater salience of pregnancy concerns, and discontinuation of condom use in relationships with boyfriends (as a demonstration of trust) have been widely reported (Abel & Brunton, 2005; Bauman & Berman, 2005; de Visser, 2005; Hatherall et al., 2005; Hillier, 1998; Holland et al., 1998; Lear, 1995). These remain important factors in young women's contraceptive decisions, but only one young woman stopped using condoms *simply* because she was in a relationship. For the rest, the (often joint) decision to stop use was encouraged by negative experiences of use.

Eight of the young women had experienced condom failures; slightly higher than the 27–36% failure rates reported in recent quantitative studies (Crosby, Yarber, Sanders, & Graham, 2005; Crosby et al., 2005; Hatherall et al., 2005; Sanders, Graham, Yarber, & Crosby, 2003). Although frequency of condom failure has been associated with greater risk of STIs (Crosby et al., 2005), none of the young women who experienced failure reported STIs. All also appeared to have avoided pregnancy by using emergency contraception. Higher condom failure rates (40%) have been reported among young men and women who report discomfort (including irritation, and loss of sensation and sexual pleasure) during use (Crosby et al., 2005). Such discomfort was a commonly reported reason for disliking condoms among the young women in this study, and it is striking how many talked of this.

Female dislike of condoms is infrequently reported in the literature (Crosby et al., 2005; Gavey, McPhillips, & Doherty, 2001; Hammer, Fisher, Fitzgerald, & Fisher, 1996; HEA, 1999). The focus instead is generally in terms of male sexual pleasure (Browne & Minichiello, 1994; Holland et al., 1998; Measor, 2006). The descriptions of condom dislike among those in this study were certainly similar to those used by men, and centred on how condoms reduced sensations, ruined the moment, and were difficult to use (Flood, 2003), but the young women were talking about their *own* sexual pleasure, and their *own* enjoyment of sex.

Although condom discontinuation is pre-empted by negative experiences of use, these were rarely enough in themselves to lead to discontinuation because they were countered by social norms of use with new or casual partners. It is being in a relationship with a boyfriend, and the trust implicit in this, which allows the change to take place. Hence, the normalisation of condom use is intrinsically limited. Condom use is more likely when young people have discussed this prior to having sex (Coleman & Ingham, 1999a; Coleman & Ingham, 1999b; Hatherall et al., 2005; Sheeran, Abraham, & Orbell, 1999), and much focus is given to the development of condom negotiation skills in HIV/STI prevention. This alone may not be enough to increase condom use and improve the sexual health of young women (and young men) because it fails to consider the wider influences of their negative experiences. Imperfect use has been associated with a lack of confidence in using condoms (Hatherall, Ingham, Stone, & McEachran, 2007). Greater promotion of condom use skills should become a focus at distribution points. Interventions to counter dislike, method failure, and the limits of the normalisation of condom use should be included in future HIV/STI prevention efforts.
